# Supplementary material for: Cultivation Conditions of Spinach and Rocket Influence Epiphytic Growth of Listeria monocytogenes
Source: Foods. 2022 Oct 1;11(19):3056. doi: 10.3390/foods11193056 (PMC9563967; doi:10.3390/foods11193056)
Supplement: Supplementary file 1 [file foods-11-03056-s001.zip › foods-1921796-supplementary.pdf]

Supplementary Tables

Table S1: Daily average values ± standard deviations of cultivation conditions. δ is growth potential. μ is maximum growth rate.

Product	Variety	Batch	Method	Time (Days)	Max Tempera-ture (°C)	Min Tempera-ture (°C)	Rain (mm)	Sunshine (hours)
Spinach	Trumpet	1 δ	Polytunnel	35	28.6 ± 4.4	12.5 ± 2.2	NA	4.1 ± 4.3
Spinach	Trumpet	2 δ	Polytunnel	35	29.6 ± 4.2	13 ± 2.1	NA	4.1 ± 3.9
Spinach	Trumpet	3 δ	Polytunnel	35	31.6 ± 4.6	14.1 ± 2.2	NA	4.7 ± 3.9
Spinach	Trumpet	1 δ	Open-fields	42	17.9 ± 1.8	11.8 ± 1.8	4.2 ± 5.4	4.1 ± 3.8
Spinach	Trumpet	2 δ	Open-fields	42	19.4 ± 2.2	12.5 ± 2.3	4.4 ± 6.2	4.7 ± 4.1
Spinach	Trumpet	3 δ	Open-fields	56	14.5 ± 3.0	7.9 ± 3.3	2.9 ± 4.2	4.1 ± 2.8
Spinach	Cello	1 δ	Polytunnel	46	27.1 ± 8.0	5.6 ± 4.3	NA	4.0 ± 3.6
Spinach	Trumpet	1 μ	Polytunnel	42	33.3 ± 5.8	8.3 ± 4.2	NA	6.2 ± 3.9
Rocket	Buzz	1 δ	Polytunnel	35	28.6 ± 4.4	12.5 ± 2.2	NA	4.1 ± 4.3
Rocket	Buzz	2 δ	Polytunnel	35	29.6 ± 4.2	13 ± 2.1	NA	4.1 ± 3.9
Rocket	Buzz	3 δ	Polytunnel	35	31.6 ± 4.6	14.1 ± 2.2	NA	4.7 ± 3.9
Rocket	Buzz	1 δ	Open-fields	42	17.8 ± 2.0	11.1 ± 1.7	3.5 ± 5.1	3.9 ± 4.1
Rocket	Buzz	2 δ	Open-fields	42	17.9 ± 1.8	11.8 ± 1.8	4.2 ± 5.4	4.1 ± 3.8
Rocket	Buzz	3 δ	Open-fields	42	19.4 ± 2.2	12.5 ± 2.3	4.4 ± 6.2	4.7 ± 4.1
Rocket	Esmee	1 δ	Polytunnel	46	27.1 ± 8.0	5.6 ± 4.3	NA	4.0 ± 3.6
Rocket	Buzz	1 μ	Polytunnel	42	33.3 ± 5.8	8.3 ± 4.2	NA	6.2 ± 3.9
Kale	Nero di Toscana	1 δ	Polytunnel	42	33.3 ± 5.8	8.3 ± 4.2	NA	6.2 ± 3.9

Table S2: Day 0 pH and water activities. Outcome in terms of effect on growth of *L. monocytogenes*. δ is growth potential. μ is maximum growth rate. NS means not significant.

Produce	Variety	Batch	Method	pH	Water Activities	Outcome
Spinach	Trumpet	1 δ	Polytunnel	7.15, 7.04, 7.02, 6.94	0.978, 0.981, 0.973, 0.984	NS
Spinach	Trumpet	2 δ	Polytunnel	7.08, 7.13, 7.21, 7.07	0.979, 0.984, 0.977, 0.976	NS
Spinach	Trumpet	3 δ	Polytunnel	6.96, 7.02, 7.03, 6.90	0.979, 0.966, 0.977, 0.981	NS
Spinach	Trumpet	1 δ	Open-fields	7.03, 7.00, 7.08, 7.13	0.971, 0.973, 0.984, 0.974	NS
Spinach	Trumpet	2 δ	Open-fields	6.94, 6.88, 7.03, 6.97	0.983, 0.967, 0.971, 0.976	NS
Spinach	Trumpet	3 δ	Open-fields	6.86, 6.92, 6.90, 6.84	0.982, 0.981, 0.979, 0.974	NS
Spinach	Cello	1 δ	Polytunnel	6.98, 6.94, 7.06, 6.98	0.976, 0.975, 0.970, 0.971	NS
Spinach	Trumpet	1 μ	Polytunnel	7.03, 7.09, 6.98, 7.13	0.992, 0.984, 0.977, 0.971	NS
Rocket	Buzz	1 δ	Polytunnel	6.56, 6.51, 6.66, 6.63	0.987, 0.983, 0.972, 0.980	NS
Rocket	Buzz	2 δ	Polytunnel	6.71, 6.56, 6.64, 6.68	0.987, 0.982, 0.988, 0.976	NS
Rocket	Buzz	3 δ	Polytunnel	6.46, 6.59, 6.61, 6.48	0.978, 0.982, 0.975, 0.981	NS
Rocket	Buzz	1 δ	Open-fields	6.61, 6.64, 6.79, 6.72	0.992, 0.984, 0.983, 0.974	NS
Rocket	Buzz	2 δ	Open-fields	6.53, 6.67, 6.66, 6.59	0.990, 0.993, 0.987, 0.982	NS
Rocket	Buzz	3 δ	Open-fields	6.54, 6.63, 6.69, 6.64	0.982, 0.979, 0.987, 0.982	NS
Rocket	Esmee	1 δ	Polytunnel	6.53, 6.49, 6.54, 6.50	0.991, 0.982, 0.984, 0.979	NS
Rocket	Buzz	1 μ	Polytunnel	6.44, 6.57, 6.60, 6.53	0.981, 0.983, 0.976, 0.975	NS
Kale	Nero di Toscana	1 δ	Polytunnel	6.52, 6.78, 6.75, 6.63	0.986, 0.991, 0.987, 0.993	NS

Table S3: Day 2, 5, 7 and 9 average pH ± standard deviations. δ is growth potential.

Produce	Variety	Batch	Method	pH Day 2	pH Day 5	pH Day 7	pH Day 9
Spinach	Trumpet	1 δ	Polytunnel	7.01 ± 0.06	7.06 ± 0.05	6.99 ± 0.04	6.98 ± 0.08
Spinach	Trumpet	2 δ	Polytunnel	7.09 ± 0.07	7.00 ± 0.11	7.05 ± 0.06	7.01 ± 0.08
Spinach	Trumpet	3 δ	Polytunnel	7.01 ± 0.06	7.04 ± 0.06	7.04 ± 0.04	7.04 ± 0.07
Spinach	Trumpet	1 δ	Open-fields	6.86 ± 0.06	6.90 ± 0.09	6.97 ± 0.11	6.97 ± 0.11
Spinach	Trumpet	2 δ	Open-fields	7.09 ± 0.11	7.03 ± 0.08	6.92 ± 0.05	6.86 ± 0.10
Spinach	Trumpet	3 δ	Open-fields	6.90 ± 0.06	6.98 ± 0.05	6.99 ± 0.10	6.90 ± 0.03
Spinach	Cello	1 δ	Polytunnel	6.84 ± 0.03	6.89 ± 0.02	6.85 ± 0.06	6.82 ± 0.07
Rocket	Buzz	1 δ	Polytunnel	6.55 ± 0.04	6.64 ± 0.05	6.68 ± 0.03	6.65 ± 0.05
Rocket	Buzz	2 δ	Polytunnel	6.58 ± 0.06	6.70 ± 0.03	6.60 ± 0.06	6.68 ± 0.06
Rocket	Buzz	3 δ	Polytunnel	6.55 ± 0.05	6.66 ± 0.07	6.67 ± 0.06	6.69 ± 0.09
Rocket	Buzz	1 δ	Open-fields	6.63 ± 0.11	6.60 ± 0.07	6.67 ± 0.06	6.78 ± 0.08
Rocket	Buzz	2 δ	Open-fields	6.65 ± 0.06	6.70 ± 0.08	6.58 ± 0.06	6.76 ± 0.05
Rocket	Buzz	3 δ	Open-fields	6.62 ± 0.06	6.59 ± 0.06	6.57 ± 0.07	6.71 ± 0.10
Rocket	Esmee	1 δ	Polytunnel	6.48 ± 0.02	6.61 ± 0.07	6.63 ± 0.05	6.67 ± 0.03
Kale	Nero di Toscana	1 δ	Polytunnel	6.70 ± 0.06	6.79 ± 0.06	6.68 ± 0.07	6.76 ± 0.05

Table S4: Day 2, 5, 7 and 9 average water activities ± standard deviations. δ is growth potential.

Produce	Variety	Batch	Method	Aw Day 2	Aw Day 5	Aw Day 7	Aw Day 9
Spinach	Trumpet	1 δ	Polytunnel	0.979 ± 0.007	0.981 ± 0.009	0.981 ± 0.008	0.977 ± 0.004
Spinach	Trumpet	2 δ	Polytunnel	0.975 ± 0.011	0.978 ± 0.004	0.982 ± 0.006	0.977 ± 0.005
Spinach	Trumpet	3 δ	Polytunnel	0.984 ± 0.005	0.977 ± 0.004	0.984 ± 0.005	0.981 ± 0.007
Spinach	Trumpet	1 δ	Open-fields	0.976 ± 0.007	0.981 ± 0.007	0.971 ± 0.001	0.974 ± 0.006
Spinach	Trumpet	2 δ	Open-fields	0.985 ± 0.005	0.976 ± 0.008	0.977 ± 0.005	0.979 ± 0.003
Spinach	Trumpet	3 δ	Open-fields	0.983 ± 0.004	0.983 ± 0.005	0.970 ± 0.002	0.978 ± 0.006
Spinach	Cello	1 δ	Polytunnel	0.979 ± 0.007	0.981 ± 0.009	0.980 ± 0.008	0.982 ± 0.002
Rocket	Buzz	1 δ	Polytunnel	0.985 ± 0.006	0.985 ± 0.003	0.976 ± 0.006	0.979 ± 0.004
Rocket	Buzz	2 δ	Polytunnel	0.977 ± 0.007	0.984 ± 0.005	0.975 ± 0.010	0.976 ± 0.011
Rocket	Buzz	3 δ	Polytunnel	0.981 ± 0.004	0.977 ± 0.010	0.977 ± 0.012	0.983 ± 0.006
Rocket	Buzz	1 δ	Open-fields	0.982 ± 0.003	0.983 ± 0.006	0.982 ± 0.008	0.986 ± 0.005
Rocket	Buzz	2 δ	Open-fields	0.979 ± 0.004	0.980 ± 0.005	0.982 ± 0.002	0.983 ± 0.006
Rocket	Buzz	3 δ	Open-fields	0.981 ± 0.003	0.983 ± 0.001	0.983 ± 0.006	0.985 ± 0.006
Rocket	Esmee	1 δ	Polytunnel	0.987 ± 0.004	0.979 ± 0.004	0.986 ± 0.006	0.982 ± 0.005
Kale	Nero di Toscana	1 δ	Polytunnel	0.988 ± 0.004	0.989 ± 0.004	0.985 ± 0.008	0.982 ± 0.003

Table S5: ComBase results based linear model of *L. monocytogenes* growth curves from growth potential experiments. δ is growth potential. μ is maximum growth rate.

Product	Variety	Batch	Method	Model	R^2	RMSE	μmax
Spinach	Trumpet	1 δ	Polytunnel	Linear	0.928	0.165	0.0157
Spinach	Trumpet	2 δ	Polytunnel	Linear	0.806	0.223	0.0123
Spinach	Trumpet	3 δ	Polytunnel	Linear	0.570	0.390	0.0129
Spinach	Trumpet	1 δ	Open-fields	Linear	0.871	0.361	0.0251
Spinach	Trumpet	2 δ	Open-fields	Linear	0.914	0.300	0.0260
Spinach	Trumpet	3 δ	Open-fields	Linear	0.951	0.140	0.0164
Spinach	Cello	1 δ	Polytunnel	Linear	0.905	0.231	0.0190
Rocket	Buzz	1 δ	Polytunnel	Linear	0.984	0.0679	0.0141
Rocket	Buzz	2 δ	Polytunnel	Linear	0.895	0.164	0.0128
Rocket	Buzz	3 δ	Polytunnel	Linear	0.949	0.153	0.0175
Rocket	Buzz	1 δ	Open-fields	Linear	0.957	0.0763	0.0095
Rocket	Buzz	2 δ	Open-fields	Linear	0.917	0.114	0.0101
Rocket	Buzz	3 δ	Open-fields	Linear	0.934	0.141	0.0140
Rocket	Esmee	1 δ	Polytunnel	Linear	0.975	0.0823	0.0136
Kale	Nero di Toscana	1 δ	Polytunnel	Linear	0.988	0.109	0.0258

Table S6: ComBase results based on best fit models of *L. monocytogenes* growth curves from growth potential experiments. δ is growth potential. μ is maximum growth rate.

Product	Variety	Batch	Method	Model	R^2	RMSE	μmax	Lag
Spinach	Trumpet	1 δ	Polytunnel	Baranyi and Roberts (no lag)	0.982	0.0829	0.0220	-
Spinach	Trumpet	2 δ	Polytunnel	Linear	0.806	0.223	0.0123	-
Spinach	Trumpet	3 δ	Polytunnel	Baranyi and Roberts (no lag)	0.999	0.0153	0.0520	-
Spinach	Trumpet	1 δ	Open-fields	Baranyi and Roberts (no lag)	0.966	0.184	0.0426	-
Spinach	Trumpet	2 δ	Open-fields	Baranyi and Roberts (no asymptote)	0.960	0.205	0.0403	81.567
Spinach	Trumpet	3 δ	Open-fields	Linear	0.951	0.140	0.0164	-
Spinach	Cello	1 δ	Polytunnel	Linear	0.905	0.231	0.0190	-
Rocket	Buzz	1 δ	Polytunnel	Baranyi and Roberts (no lag)	0.985	0.0661	0.0151	-
Rocket	Buzz	2 δ	Polytunnel	Linear	0.895	0.164	0.0128	-
Rocket	Buzz	3 δ	Polytunnel	Biphasic (no lag)	0.980	0.0971	0.0202	-
Rocket	Buzz	1 δ	Open-fields	Linear	0.957	0.0763	0.0095	-
Rocket	Buzz	2 δ	Open-fields	Linear	0.917	0.114	0.0101	-
Rocket	Buzz	3 δ	Open-fields	Linear	0.934	0.141	0.0140	-
Rocket	Esmee	1 δ	Polytunnel	Linear	0.975	0.0823	0.0136	-
Kale	Nero di Toscana	1 δ	Polytunnel	Baranyi and Roberts (no lag)	0.990	0.101	0.0276	-

Table S7: Maximum growth rates of *L. monocytogenes* strains 959 and 1382 on experimental farm Spinach (F1 Trumpet) and Rocket (Buzz)

**Product**	**Strain**	**Model**	**R^2**	**RMSE**	**Maximum growth rate (μmax) Ln cfu g^{-1} h^{-1}**
Spinach	959	Linear	0.836	0.193	**0.0127**
Spinach	959	Baranyi & Roberts (no lag)	0.942	0.115	**0.0211**
Spinach	959	Biphasic model (no lag)	0.950	0.106	**0.0197**
Spinach	1382	Linear	0.916	0.142	**0.0136**
Spinach	1382	Baranyi & Roberts (no lag)	0.934	0.125	**0.0158**
Spinach	1382	Biphasic (no lag)	0.946	0.114	**0.0157**
Rocket	959	Linearǂ	0.972	0.0935	**0.0160**
Rocket	1382	Linear	0.950	0.117	**0.0147**
Rocket	1382	Baranyi & Roberts (no lag)	0.949	0.118	**0.0162**
Rocket	1382	Biphasic (no lag)	0.943	0.124	**0.0148**

ǂ *Only the linear model could be fitted in Combase*

Table S8: ComBase results based linear model of TBC growth curves from growth potential experiments. δ is growth potential.

Product	Variety	Batch	Method	Model	R^2	RMSE	μmax
Spinach	Trumpet	1 δ	Polytunnel	Linear	0.700	0.285	0.0120
Spinach	Trumpet	2 δ	Polytunnel	Linear	0.731	0.263	0.0119
Spinach	Trumpet	3 δ	Polytunnel	Linear	0.911	0.128	0.0109
Spinach	Trumpet	1 δ	Open-fields	Linear	0.682	0.246	0.0100
Spinach	Trumpet	2 δ	Open-fields	Linear	0.410	0.374	0.0095
Spinach	Trumpet	3 δ	Open-fields	Linear	0.707	0.354	0.0152
Spinach	Cello	1 δ	Polytunnel	Linear	0.906	0.275	0.0228
Rocket	Buzz	1 δ	Polytunnel	Linear	0.667	0.368	0.0145
Rocket	Buzz	2 δ	Polytunnel	Linear	0.899	0.224	0.0178
Rocket	Buzz	3 δ	Polytunnel	Linear	0.911	0.278	0.0237
Rocket	Buzz	1 δ	Open-fields	Linear	0.941	0.223	0.0237
Rocket	Buzz	2 δ	Open-fields	Linear	0.758	0.386	0.0187
Rocket	Buzz	3 δ	Open-fields	Linear	0.965	0.126	0.0176
Rocket	Esmee	1 δ	Polytunnel	Linear	0.934	0.208	0.0207
Kale	Nero di Toscana	1 δ	Polytunnel	Linear	0.991	0.0774	0.0212

Table S9: ComBase results based on best fit models of TBC growth curves from growth potential experiments. δ is growth potential.

Product	Variety	Batch	Method	Model	R^2	RMSE	μmax	Lag
Spinach	Trumpet	1 δ	Polytunnel	Biphasic (no lag)	0.971	0.088	0.0216	-
Spinach	Trumpet	2 δ	Polytunnel	Baranyi and Roberts (no lag)	0.858	0.191	0.0361	-
Spinach	Trumpet	3 δ	Polytunnel	Baranyi and Roberts (no lag)	0.978	0.0632	0.0154	-
Spinach	Trumpet	1 δ	Open-fields	Baranyi and Roberts (no asymptote)	0.985	0.0527	0.0288	136.112
Spinach	Trumpet	2 δ	Open-fields	Biphasic (no lag)	0.962	0.0954	0.0499	-
Spinach	Trumpet	3 δ	Open-fields	Biphasic (no lag)	0.962	0.128	0.0269	-
Spinach	Cello	1 δ	Polytunnel	Baranyi and Roberts (complete)	0.999	0.0267	0.0398	85.974
Rocket	Buzz	1 δ	Polytunnel	Baranyi and Roberts (no asymptote)	0.946	0.148	0.0513	150.872
Rocket	Buzz	2 δ	Polytunnel	Linear	0.899	0.224	0.0178	-
Rocket	Buzz	3 δ	Polytunnel	Linear	0.911	0.278	0.0237	-
Rocket	Buzz	1 δ	Open-fields	Baranyi and Roberts (no asymptote)	0.944	0.0219	0.0317	63.244
Rocket	Buzz	2 δ	Open-fields	Linear	0.758	0.386	0.0187	-
Rocket	Buzz	3 δ	Open-fields	Baranyi and Roberts (no asymptote)	1	0.0124	0.0220	49.266
Rocket	Esmee	1 δ	Polytunnel	Baranyi and Roberts (no lag)	0.998	0.0399	0.0278	-
Kale	Nero di Toscana	1 δ	Polytunnel	Baranyi and Roberts (complete)	1	0.0003 04	0.0244	27.28
